# High-grade hyperinvasive sarcomatoid urothelial bladder carcinoma demonstrating complete response to bladder-preserving chemoradiation

**DOI:** 10.3747/co.v16i3.396

**Published:** 2009-05

**Authors:** J.B. Wallach, B. Wang, N. Sanfilippo

**Keywords:** Bladder carcinoma, sarcomatoid carcinoma, chemotherapy, radiation oncology, cystectomy, quality of life, bladder preservation

## Abstract

The standard treatment for locally advanced urothelial bladder carcinoma is radical cystectomy or chemoradiation. Sarcomatoid urothelial carcinoma, a rare tumour, is treated with radical cystectomy because the response to radiation therapy alone is poor in other sarcomas. We report a case of high-grade hyperinvasive urothelial bladder carcinoma with sarcomatoid differentiation. The patient refused cystectomy, and so a chemoradiation regimen was devised for her treatment. She completed the regimen and has since demonstrated a complete response to chemoradiation therapy clinically and pathologically by biopsy. The patient has therefore been able to attain a complete response while preserving a functional bladder.

## 1. CASE DESCRIPTION

**FIGURE 1:**
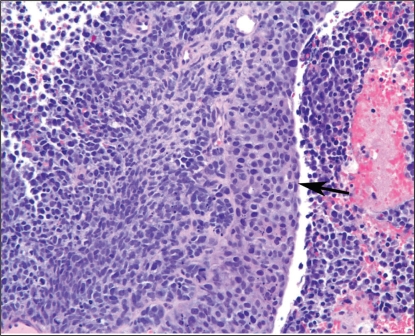
Invasive high-grade urothelial carcinoma (arrow) with prominent sarcomatoid and neuroendocrine elements.

Presentation of a 67-year-old white woman who had experienced several weeks of hematuria with clots led to consultations at two outside hospitals (oshs). Cystoscopy and transurethral resection at the first osh revealed a high-grade urothelial bladder carcinoma with areas of sarcomatoid and neuroendocrine differentiation. The patient was advised to have radical cystectomy. A repeat biopsy at the second osh revealed invasive high-grade urothelial carcinoma with prominent sarcomatoid and neuroendocrine elements, and invasion of the muscularis propria, classified as T2AN0 stage ii (Figure 1). The patient was further advised that bladder-conserving approaches were not recommended because of the likely poor response to chemoradiation by the sarcomatoid elements, rendering radical cystectomy the standard of care. However, the woman continued to seek bladder preservation and presented to our institution to discuss bladder-sparing options. We also advised her that radical cystectomy was the traditional standard of care, but agreed that chemoradiation was a reasonable alternative because cystectomy could be reserved for salvage.

Clinical examination revealed no significant physical findings, and the patient denied further hematuria, increased urinary frequency, urinary urgency, urinary retention, dysuria, incontinence, anorexia, or weight loss. She denied ever smoking, but reported a significant history of second-hand exposure. Her past medical history was significant for a peptic ulcer, and her past surgical history included appendectomy and tonsillectomy.

We developed a treatment program consisting of 7 weeks of weekday radiation therapy integrated with chemotherapy, with follow-up routine cystoscopy thereafter to monitor response. The patient’s radiation program consisted of 36 fractions over 49 days, with a dose of 39.6 Gy to the bladder and pelvic lymph nodes followed by 25.2 Gy to the bladder tumour. Her chemotherapy regimen consisted of cisplatin 35 mg/m^2^ (49 mg in 500 mL normal saline over 1 hour) at the beginning of weeks 1, 3, 5, and 7, with each cycle divided over 2 consecutive days. She was also treated with palonosetron HCl 0.25 mg intravenous (IV) bolus and dexamethasone 20 mg IV in 50 mL normal saline while receiving chemotherapy.

**FIGURE 2:**
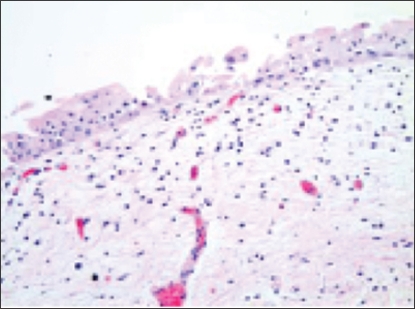
Post-chemoradiation treatment biopsy, showing benign urothelial mucosa without residual tumour.

The patient tolerated therapy well, without interruptions and with only mild diarrhea that improved with loperamide. Cystoscopy was performed 2.5 months after chemoradiation and revealed no cancerous lesions. Biopsies taken from the initial tumour location at that time were negative for carcinoma (Figure 2).

## 2. DISCUSSION AND CONCLUSIONS

Bladder cancer is the most common urinary tract malignancy, with 67,160 new cases and 13,750 deaths occurring each year in the United States 1. The 3:1 male predominance is believed to be a result of higher smoking rates and greater exposure to occupational poisons among men, although some researchers postulate that the more-active androgen receptor in men accelerates carcinogenesis 2–4. Overall, the 5-year survival rate is 82% 1.

In North America and Europe, simple urothelial carcinomas account for more than 90% of bladder malignancies, with epithelial tumours constituting an additional 9% 5,6. The treatment protocol depends on the depth of invasion, which is categorized as superficial, muscle-invasive, or hyperinvasive. Superficial malignancies may be treated with electrocautery or instillation of bacille Calmette–Guérin; hyperinvasive tumours necessitate radical cystectomy with chemoradiation. For muscle-invasive tumours, radical cystectomy with or without chemotherapy has been the traditional standard, but in an effort to improve quality of life by avoiding urinary diversion procedures, the bladder-sparing trimodality therapy of transurethral resection of bladder tumour, systemic chemotherapy, and concurrent radiation therapy has increasingly been used in selected patients. Several studies have concluded that this approach has a 5-year survival for muscle-invasive tumours comparable to that for radical cystectomy in patients of similar age and stage (generally 40%–60%) 7–10.

Sarcomatoid carcinomas of the bladder are exceedingly rare and highly aggressive spindle-cell neoplasms that are usually diagnosed at an advanced stage and have a median survival of 10 months 7,11. Radical surgery with adjuvant radiation therapy to eradicate microscopic disease is the traditional standard treatment for most sarcomas, because these tumours show poor response to primary radiation therapy 7. Literature for nonsurgical therapy for these bladder tumours is scarce. One case report describes a patient with metastatic sarcomatoid bladder carcinoma who demonstrated a clinical complete remission after cisplatin and gemcitabine, and another describes a complete remission after neoadjuvant chemotherapy using carboplatin and gemcitabine followed by partial cystectomy 12,13. Our report demonstrates only 1 case with limited follow-up, but the favourable tumour response, coupled with an improved quality of life with a native bladder, provides a strong argument for bladder-preserving chemoradiation as an alternative treatment regimen.
